# Self-Reported Prevalence of Allergies in the USA and Impact on Skin—An Epidemiological Study on a Representative Sample of American Adults

**DOI:** 10.3390/ijerph17103360

**Published:** 2020-05-12

**Authors:** Sophie Seité, Alyce Mei-Shiuan Kuo, Charles Taieb, Tamara Lazić Strugar, Peter Lio

**Affiliations:** 1 La Roche-Posay Dermatological Laboratories, 92300 Levallois-Perret, France; 2 Icahn School of Medicine at Mount Sinai, New York, NY 10029, USA; alyce.kuo@icahn.mssm.edu (A.M.-S.K.); tamara.lazic@aya.yale.edu (T.L.S.); 3 European Market Maintenance Assessment, 94120 Fontenay-sous-Bois, France; charles.taieb@emma.clinic; 4 Medical Dermatology Associates of Chicago, Chicago, IL 60654, USA; peterlio@gmail.com

**Keywords:** allergies, food allergy, skin allergy, respiratory allergy, prevalence, skin side effects

## Abstract

Background: The rising prevalence of allergies can substantially impact the skin, which is one of the largest targets for allergic and immunologic responses. Objective: Here, we describe the results of an online survey assessing self-reported allergy prevalence in Americans, outline the populations who report allergies, and characterize the skin conditions associated with allergy. Methods: An online survey was conducted in the USA of 2008 adults as a representative sample of the general American population. Results: 41.7% of American adults (mean age 44.7 ± 15.3 years old) reported having allergies. Reported allergies included respiratory allergies (45.2%), skin allergies (41.4) and food allergies (33.9%). 47.7% of those who reported allergies also reported experiencing associated skin reactions. In addition, those who reported allergies were 2 to 4.5 times more likely to report a cutaneous skin disease, 7 times more likely to report sensitive skin, and twice as likely to report experiencing skin reactions when using skincare products compared to those who did not report allergies. Conclusions: It is estimated that over 100 million American adults have allergies. These results will help raise awareness about the burden of allergies and the need to develop solutions to mitigate their impact on health.

## 1. Introduction

In the Western world, the prevalence of allergies, including hay fever, asthma, and especially now food allergy, has been on the rise, a phenomenon referred to as the “allergy epidemic” [1]. For instance, in children, hospital admissions due to food anaphylaxis doubled in the United States between 2000 and 2009 [2,3].

The term “allergy” is broad and can refer to many types of food, respiratory, and skin reactions. In the United States, common food allergens include milk, eggs, soy, and peanuts, with allergy symptoms ranging from hives, coughing, or vomiting, to difficulty breathing and loss of consciousness. Although food allergies are most common in children, many adults continue to face food allergies developed in childhood and can also develop new ones [4]. Respiratory allergies include allergic rhinitis and allergic asthma, which share many features and are frequently associated with air pollution and pollens [5,6]. The haptens involved in skin allergies are diverse and can include preservatives, fragrances, and metals [7]. While these allergies may present with varied symptoms, they share immunologic foundations and pathways. This is especially evident in processes such as atopic march, where in infancy, the development of atopic dermatitis, a skin condition, can lead to food allergies as well as allergic rhinitis and asthma later in life [8].

To diagnose clinically, allergies can require tests such as oral food challenge or patch testing, which provoke symptoms, making the prevalence of allergy difficult to estimate. Nonetheless, it is necessary to make this assessment to better understand the tremendous impact of allergies. Here, we describe the results of an online survey assessing self-reported allergy prevalence in Americans, outline the populations who report allergies, and characterize the skin conditions associated with allergy.

## 2. Materials and Methods

### 2.1. Study Population

A polling institute (HC Conseil Paris, France) conducted the current survey between December 2018 and January 2019. A sample of the general US adult population, over 18 years of age, was recruited. Proportional quota sampling was applied to render the study population representative of the US general adult population following data available and published (U.S. Census Bureau, June 2017). These quotas were based on the following aspects: sex, age, socio-professional status, and regional distribution (Table 1).

Data were collected via Internet by random selection of 2008 US people among the large number of internet users over 18 years of age who agreed to participate. Each participant was contacted by e-mail, and if the contact failed or questionnaire was not entirely completed, another participant with the same characteristics was randomly selected. Missing data were not allowed and respondents were required to provide an answer to all questions.

### 2.2. Survey

This research employed completely anonymized data without involving direct patient contact, and institutional review board approval was not necessary prior to study initiation. Respondents were asked a range of sociodemographic questions including gender, age, occupation, social class, area of residence, and tobacco use. They were also asked questions about skin phototype, occurrence of allergies, types of allergies, allergens, medical diagnosis confirmation, therapeutic treatment, symptoms, skin pathologies, skin effects, and skin symptoms. Questions regarding the impact of environmental factors such as exposure to environmental pollution and sun were also asked.

### 2.3. Statistical Analysis

In this descriptive study, participants who reported allergies were compared to participants who did not report any allergies. Quantitative variables were expressed as mean and standard deviation. Qualitative variables were expressed as frequencies and percentages. Comparisons between groups were performed using the Student test in the case of quantitative variables; for categorical variables, intergroup comparisons were done with the *χ^2^* test. Relative risk (RR) was calculated for comparison of the population who reported allergies to the population who did not reported allergies. The level of significance was set at 5%. Statistical analyses were performed using R software version 3.6.1 (Vienna, Austria).

## 3. Results

### 3.1. General Population

Of the 2008 respondents (18–74 years, 49% men, 51% women), 41.7% of participants (mean age 44.7 ± 15.3 years) reported having allergies (44.6% men, 55.4% women). Of the total population, 25.4% lived in rural areas (≤50,000 residents), 48.9% in suburban or medium size cities (50,000–1,000,000 residents), and 25.7% in large cities (≥1,000,000 residents), and 25% were smokers. The phototype distribution of the total population was 21.1%, 23%, 35.5%, 12.5%, 4.3%, and 3.6% for phototypes I to VI, respectively.

Reported allergies included respiratory allergies (45.2%), skin allergies (41.4%), and food allergies (33.9%). Of the total, 68.4% reported their allergies had been diagnosed by at least one doctor, who most frequently was either a general practitioner, allergy specialist, or dermatologist (Table 2). However, many with allergies reported not using any treatments such as corticosteroids or antihistamines—29.8%, 46.1%, and 55.6% of those with respiratory, skin, and food allergies, respectively.

Only 39.7% were able to identify the allergen(s) responsible for their allergies (mainly pollens, mites, and mold), as well as the main symptoms associated with their allergies, such as allergic rhinitis or asthma (Table 3).

Of those who reported allergies, 47.7% also reported experiencing associated skin reactions. In 47.2% of these cases, a doctor diagnosed this skin reaction, and 37% reported using topical and/or oral treatments (Table 4).

### 3.2. Allergic Population Versus Non-Allergic Population

The population who reported allergies was slightly older (mean age 44.7 ± 15.3 vs 43.1 ± 15.7 years, *p* < 0.00307) than the population who did not report allergies. The population with allergies also included significantly more women (55.4% vs 48%, *p* = 0.0011). However, the two populations were similar in distributions regarding their geography, skin phototypes, and smoking statuses.

**Figure 1:**
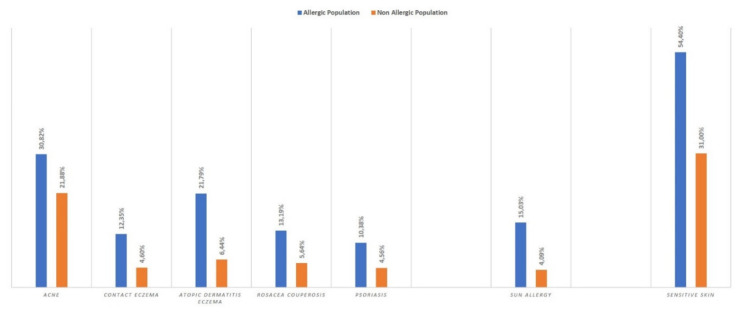
Skin diseases in the two populations.

Those who reported allergies were 1.4 to 3.7 times more likely to also report a skin disease such as eczema or atopic dermatitis (RR = 3.39 [2.60–4.39]), sun allergy (RR = 3.68 [2.64–5.11]), contact eczema (RR = 2.68 [1.93–3.72]), psoriasis (RR = 2.28 [1.62–3.18]), rosacea/couperosis (RR = 2.34 [1.73–3.51]), or acne (RR = 1.41 [1.21–1.63]), and close to 2 times more likely to report sensitive skin (RR = 1.7 [1.57–1.95]) compared to those who did not report any allergies (Figure 1).

They were significantly more likely to report sensitive skin (54.3% vs 31.0%), particularly very sensitive skin (18.7 vs 6%, *p* < 0.0001), sensitive eyes (53.9% vs 25.7%, *p* < 0.00006), and having parents with sensitive skin (33% vs 17.7%, *p* < 0.0001). Notably, 24.7% of those who reported allergies also reported having atopic dermatitis during childhood compared to only 9.9% in those who did not report any allergies (*p* < 0.00001).

**Figure 2:**
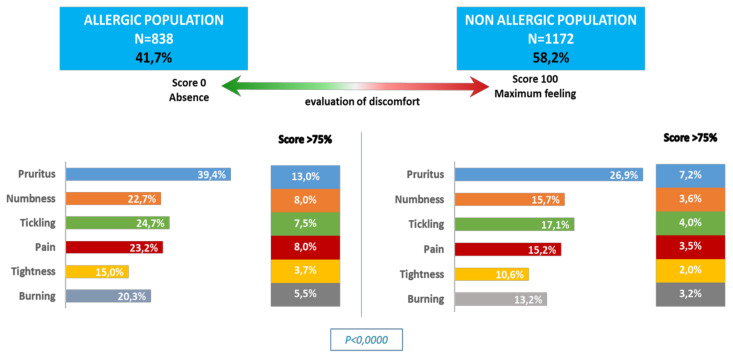
Skin discomforts in the two populations.

**Figure 3:**
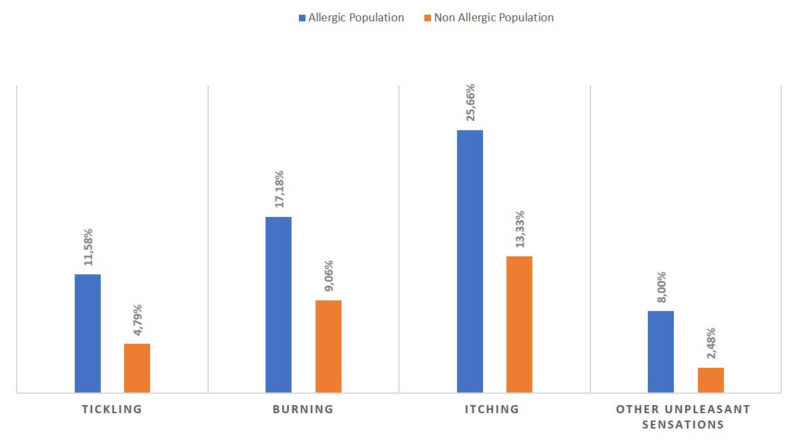
Skin discomforts associated with skincare products in the two populations.

Additionally, those who reported allergies were more likely to experience skin discomfort and reported a higher incidence of severe skin discomfort (Figure 2). They were also more likely to report experiencing skin reactions (pruritus: RR = 1.92; burning: RR = 1.90; tickling: RR = 2.42 *p* < 0.001) when using skincare products (Figure 3).

### 3.3. Environmental Impact

The population who reported allergies was significantly more impacted by air, water, ground, noise, light, and radiation pollution (*p* < 0.0001) than the population who did not report allergies (Table 5). They more frequently claimed that pollution affected their way of life (53.6% vs 34.5%, *p* < 0.0001) and impacted their health and well-being (71.1% vs 53.2%, *p* < 0.0001). They also more commonly noted an impact of pollution on their skin (29.5% vs 17.8%, *p* < 0.002). In those with allergies, 32.6% reported that the impact pollution made on their skin was quite or very important compared to 17.9% in those without allergies (*p* < 0.0001). Those with allergies were also more likely to use skincare products to protect their skin against pollution (5% vs 2.8%, *p* < 0.0001).

A significantly larger part of those with allergies had moderate and intense daylight solar exposure than in those who did not report allergies (51.8% vs 39.8%, *p* < 0.0004). Nonetheless, only 19% of those with allergies reported not using any photoprotection in comparison to 28.5% of those without allergies (*p* < 0.0001). Those with allergies were also more likely to apply sunscreen during outdoor activities (37.4% vs 27.9%, *p* < 0.0001), when working outdoors (34.2% vs 27%, NS), and during intense sun exposure (52.5% vs 48.4%, NS).

## 4. Discussion

In this self-reported survey of a representative sample of the general American population, 41.7% of survey respondents reported having allergies. This figure closely resembles the results of an earlier study of allergic sensitization using data from the National Health and Nutrition Examination Survey (NHANES) 2005–2006, which found that 44.6% of the sampled population tested positive to at least one specific allergen based on serum IgE testing [9]. However, while the NHANES data showed that prevalence of allergic sensitization was higher in men, this survey found that self-reported allergy was more prevalent in women. The survey results parallel research showing that due to a combination of societal and biological factors, women tend to have poorer self-reported health [10]. One possible explanation for this discrepancy is that in general, women tend to be more health conscious [11]. It is possible that although men have higher rates of allergen sensitization, women are more likely to notice the allergy symptoms resulting from sensitization and self-report it.

The self-perceived diagnosis or symptoms of allergies may be one limitation of this study, as only 68.4% of the respondents who reported allergies said that these allergies had been officially diagnosed by a doctor. This can be problematic because participants frequently overreport symptoms of allergic disease, and a non-immunologically based adverse response to a food may easily be misconstrued to be an allergic reaction and self-reported as such [12]. Although assessing clinical response to allergens, such as through food challenge, is more objective, it can also put patients in danger. Thus, accurate allergy prevalence can be challenging to estimate [13]. Another limitation of this study is that only adults 18 years and older were sampled when allergy rates are increasing most rapidly among children [3].

There are many theories attempting to explain the ongoing escalation in allergy prevalence, one of which is the hygiene hypothesis. The hygiene hypothesis proposes that decreased exposure to infections has led to increased immune response to allergens. It postulates that IgE antibodies, which defend the body against parasitic infections, may be redirected toward less offensive environmental allergens such as pollen, generating allergies [14]. Although improved hygiene may indeed play a role, other lifestyle changes in westernized countries such as decreased physical activity, shifts in diet to include more processed foods, and changes in daily ways of living likely make an impact as well. For example, increased bathing of babies can alter skin permeability, leading to increased allergic sensitization [15,16].

The role of the skin barrier in allergic sensitization has been well-described. Specifically, dysfunction of the skin barrier can increase the likelihood of allergens coming into contact with the immune system, which can trigger sensitization [17]. However, the impact of allergies on other skin conditions has been less thoroughly characterized. Nonetheless, the survey results presented here show a clear association between reporting any type of allergy and also reporting a skin disease or skin sensitivity. While some of these links are relatively well-established, such as that between food allergy and atopic dermatitis, others are less clear [18]. Emerging research is beginning to look into these associations, such as the recent discovery of a shared IL-17 immunophenotype between psoriasis and asthma, but we are far from having a complete understanding of the relationship between various types of allergies and skin diseases [19].

Understanding allergy is critical to providing care to the vast proportion of Americans who suffer from its symptoms. For the millions of people coping with allergies, allergies can create significant burden on day to day life. Anxiety, impact on relationships, embarrassment, and frequent interruptions to normal tasks brought on by respiratory, food, and skin allergy symptoms all contribute to poorer quality of life in those with allergies [20,21,22].

Much work still needs to be done in developing ways to manage allergies. For food allergies, strict avoidance of the causative food allergen has been traditionally recommended; however, there has been research into developing tolerance through oral, sublingual, or epicutaneous immunotherapy [23]. Meanwhile, respiratory allergies are frequently treated with corticosteroids, and minimizing skin barrier dysfunction may be one way to prevent skin allergies [17,24]. These approaches to management can grow taxing, as allergies are lifelong, chronic conditions. Additionally, strategies such as avoidance can be challenging, as only 39.7% of survey respondents are able to identify the causative allergens. Without well-developed solutions and cures to allergies in place, the prevalence of allergies is bound to continue to rise, even as incidence stabilizes [2].

## 5. Conclusions

With the rising prevalence of allergies in the Western world, it is increasingly important to better characterize the nature and implications of this epidemic. Until the pathogenesis is fully understood, the skin and its barrier function remain important targets for study, especially given the large number of unidentified allergens.

## Figures and Tables

**Table 1:** Quota used to select the study population.

US Sex and Age Breakdown	Males	Females
18–24	7%	7%
25–34	10%	10%
35–44	9%	9%
45–54	10%	10%
55–64	8%	9%
65–74	5%	6%
**USA Regions**		
West	23%	
Midwest	23%	
South	36%	
Northwest/Northeast	18%	
**USA Annual Income in US$**		
Less than 40,000	40%	
More than 40,000	60%	

**Table 2:** Doctors who diagnosed allergies.

	*n*	%
**Participants Reporting an Allergy**	**838**	**41.73%**
Subjects able to name the allergy	333	39.74%
**Percentage of Participants Diagnosed by A Doctor**	**573**	**68.38%**
Health professional who diagnosed the allergy		
General practitioner	259	45.20%
Allergy specialist	145	25.31%
Dermatologist	94	16.40%
Pediatrician	33	5.76%
Otolaryngologist	14	2.44%
Pulmonary specialist	10	1.75%
Acupuncturist	2	0.35%
Homeopathic doctor	1	0.17%
Another specialized physician	15	2.62%

n: number of participants.

**Table 3:** Symptoms and allergens related by the allergic population.

	*n*	%
**Symptoms Associated with Allergy Reported by Participants**		
Allergic rhinitis (hay fever)	367	43.79%
Asthma	220	26.25%
Eczema/Atopic dermatitis	188	22.43%
Bronchitis with wheezing	151	18.02%
Conjunctivitis	61	7.28%
Edema	35	4.18%
Other	195	23.27%
**Allergens Reported by Participants**	***n***	**%**
Pollens	529	63.13%
Dust mites	353	42.12%
Mold	294	35.08%
Dogs, cats, ferrets, other animals	214	25.54%
Food allergens	177	21.12%
Latex	60	7.16%
Cockroaches	59	7.04%
Hymenoptera (bees, wasps, hornets, etc.)	50	5.97%
Other	125	14.92%

n: number of participants.

**Table 4:** Skin reactions associated with allergies.

	*n*	%
**Percentage of Participants Reporting Skin Reaction**	**400**	**47.73%**
Percentage managed by a doctor	189	47.25%
**Who is the Health Professional Who Managed the Skin Reaction?**		
General practitioner	88	46.56%
Allergy specialist	59	31,22%
Dermatologist	26	13.76%
**Participants Reporting Prescribed Treatment for Skin Reaction**	**148**	**78.30%**
What kind of treatment was prescribed for your skin reaction?		
Topical	106	71.62%
Oral	66	44.59%
Skincare products	21	14.19%

n: number of participants.

**Table 5:** Impact of pollution in the two populations.

	Impacted		Worried	
	Allergic	Non-Allergic	Allergic	Non-Allergic
	*n* = 838	*n* = 1172	*n* = 838	*n* = 1172
Air	74.22%	61.28%	51.19%	34.96%
Water	29.47%	22.65%	6.56%	7.18%
Soil	12.05%	6.75%	2.86%	1.62%
Noise	35.92%	27.01%	11.22%	13.59%
Light	31.86%	22.31%	9.31%	5.98%
Radiation	18.26%	15.13%	4.06%	4.79%

n: number of participants.
